# Effect of a Soluble Epoxide Hydrolase Inhibitor, UC1728, on LPS-Induced Uveitis in the Rabbit

**DOI:** 10.13188/2334-2838.1000024

**Published:** 2016-01-12

**Authors:** Gillian J. McLellan, Zeynep Aktas, Elizabeth Hennes-Beean, Aaron W. Kolb, Inna V. Larsen, Emily J. Schmitz, Hilary R. Clausius, Jun Yang, Sung Hee Hwang, Christophe Morisseau, Bora Inceoglu, Bruce D. Hammock, Curtis R. Brandt

**Affiliations:** 1 Department of Ophthalmology and Visual Sciences, School of Medicine and Public Health, University of Wisconsin-Madison, Wisconsin, USA; 2 Department of Medical Microbiology and Immunology, School of Medicine and Public Health, University of Wisconsin-Madison, Wisconsin, USA; 3 Department of Surgical Sciences, School of Veterinary Medicine, University of Wisconsin-Madison, Wisconsin, USA; 4 Comparative Ophthalmic Research Laboratories, School of Veterinary Medicine, University of Wisconsin-Madison, Wisconsin, USA; 5 Department of Ophthalmology and Visual Sciences, McPherson Eye Research Institute, University of Wisconsin-Madison, Wisconsin, USA; 6 Department of Surgical Sciences, Gazi University, Turkey; 7 Department of Entomology and Comprehensive Cancer Center, University of California, Davis, CA 95616, USA

**Keywords:** UC1728, Uveitis, Cytochrome P450, Soluble epoxide hydrolase inhibitor, Inflammation

## Abstract

Cytochrome P450 epoxygenase isozymes convert free arachidonic acid into eicosanoids named epoxyeicosatrienoic acids (EETs) that have roles in regulating inflammation. EETs are rapidly converted to dihydroxyeicosatrienoic acids (DiHETs) by soluble epoxide hydrolase (sEH). Little is known about the potential role of these metabolites in uveitis, but conversion of EETs to DiHETs could contribute to the inflammation. We tested a potent and orally available inhibitor of sEH for its ability to reduce ocular inflammation in a rabbit LPS-induced model of uveitis. Rabbits were treated by subcutaneous injection with the sEH inhibitor (UC1728, 3 mg/kg), or the vehicle control (PEG400) and uveitis was assessed at 6, 24 and 48 h post-intracameral LPS injection using a modified Hackett-McDonald scoring system. Eyes treated by intra-cameral injection of PBS, or by aseptic preparation served as further controls. Signs of inflammation in this model were mild and transient. Treatment with UC1728 did not significantly reduce inflammation compared to animals treated with the PEG400 vehicle. Blood levels of UC1728 were a thousand fold higher than the in vitro determined inhibitory potency (IC50) of the compound suggesting a significant degree of inhibition of sEH in the rabbit. The lack of efficacy suggests that sEH or its substrates the EETs may not be involved in mediating inflammation in this model of uveitis.

## Introduction

Arachidonic acid (AA) metabolites play critical roles in a number of physiological processes, including pro-and anti-inflammatory activities, peripheral sensitization to pain, regulation of kidney function and blood pressure, protection against oxidative injury in the heart, regulation of platelet activity, and cancer [1-6]. Arachidonic acid, released from cellular membranes by phospholipase A2, is the substrate for three different pathways generating functional metabolites. Cyclooxygenases and prostaglandin synthases convert AA into prostaglandins, thromboxane, and prostacyclins. Lipoxygenases convert AA into leukotrienes, endogenous mono-hydroxy-eicosatetraenoic acids (HETEs) and lipoxins; and cytochrome P450 epoxygenases convert AA into epoxyeicosatrienoic acids (EETs). The cytochrome P450 epoxygenase-derived EETs have anti-inflammatory properties and are also analgesic and anti-convulsant lipid mediators [3,5,7-9]. These bioactive lipids are degraded by the soluble epoxide hydrolase (sEH) and converted into dihydroxyeicosatrienoic acids (DiHETs) which are thought to be pro-inflammatory. Inhibition of sEH therefore stabilizes EETs by preventing or delaying their conversion into pro-inflammatory DiHETs and preserves EETs that display anti-inflammatory effects in models of systemic inflammation such as the mouse LPS induced sepsis model.

Members of the cytochrome P450 A and B subfamilies are present in the eye, particularly in ciliary body epithelium and corneal epithelium, and can be induced by exposure to various chemicals, hypoxia and inflammatory stimuli [10-20]. Evidence that cytochrome P450 isoforms have physiological roles in the eye, come from several studies. Mutations in CYP1B/1 are causative for Primary Congenital Glaucoma (reviewed in [21]), inhibition of CYP450 by stannous chloride reduced the inflammatory response in a closed eye contact lens rabbit model [22], and administration of siRNA targeting CYP4B1 inhibited corneal neovascularization in a suture-induced rabbit model by reducing production of 12-HETrE and inhibiting vascular endothelial growth factor (VEGF) expression [23]. Thus, CYP450 enzymes play important roles in regulating inflammatory and other responses in the eye.

Soluble epoxide hydrolase has been a target for drug development for some time and a large number of compounds have been tested in various models [3,7,24-28]. DiHETs have largely been found inactive or less active than EETs in assays that used these molecules barring few exceptions [29]. The finding that linoleic metabolites, DiHOMEs (dihydroxy octadecamoenoate esters), are more toxic to the lung than their corresponding EpOME (monoepoxide derivatives of linoleic acid) precursors suggest that they may be pro-inflammatory [30].

Early sEH inhibitors suffered from poor solubility and less than ideal pharmacokinetics. More recently, orally available inhibitors with improved potency have been synthesized [31]. One compound, *trans*-4-{4-[3-(4-trifluormethoxy-phenyyl)-ureido]-cyclohexyloxy}-benzoic acid (UC1728) is a potential inhibitor of rabbit sEH with an IC_50_ of 2.0 nM. UC1728 also has a favorable pharmacokinetic profile with peak serum levels (320 nM) in mice given a dose of 1 mg/kg, occurring between 5 and 7 h post-administration with a half-life of 15 to 20 h. It was successful in reducing the severe pain and inflammation associated with laminitis in horses [32]. Thus UC1728 is an excellent “probe” to understand the roles of sEH and EETs and a potential candidate for further drug development.

Uveitis encompasses a number of ocular inflammatory conditions and accounts for 5-20% of blindness in the USA and Europe. In developing countries uveitis can account for as much as 25% of legal blindness [33,34]. Corticosteroids are commonly used to treat uveitis but not all patients respond. Immunosuppressive drugs such as cyclosporine, azathioprine, mycophenolate mofetil, and methotrexate are used as second line drugs. More recently, inhibitors that target TNF-α, such as infliximab, etanercept, and adalimumab, have been effective in patients that are refractory to other treatments [35]. Several of these therapeutic agents have unfavorable side effects or toxicity and carry the potential for serious adverse effects, thus additional therapeutic agents are needed to effectively treat uveitis. Three basic types of animal models have been used to test therapeutic agents for uveitis. One acute model involves the intra-vitreal or intra-cameral injection of bacterial endotoxin [36,37]. Another acute model involves immunizing animals and then injecting the antigen into the anterior chamber [38]. A chronic model involves immunizing animals with interphotoreceptor retinoid-binding protein (IRBP) or visual arrestin [39]. The chronic models mimic an autoimmune-mediated mechanism with T-cell involvement [35,40].

Inhibitors of the sEH have been shown to be anti-inflammatory in numerous models of acute inflammation, most prominently LPS induced systemic inflammation [41,42]. In this model inhibition of sEH increases survival at 24 h, reverses hypotension, decreases pain and decreases the levels of pro-inflammatory AA metabolites. Therefore the goal of this study was to test the hypothesis that UC1728 will be efficacious in reducing the acute inflammation induced by endotoxin in the rabbit anterior chamber model of uveitis. We found that UC1728 did not significantly reduce the severity of aqueous cell and flare or iris congestion. Mean blood level of UC1728 was 2070±196 nM 48 h after injection of UC1728 which is about a thousand fold above the in vitro IC_50_ on rabbit sEH arguing the lack of effect is not due to low drug levels. These results suggest that sEH, and by inference EETs and DiHETs do not play a significant role in the acute inflammation induced by endotoxin injection into the anterior chamber of rabbit eyes.

## Methods

### Synthesis and potency determination of sEH inhibitors

Inhibitors of sEH were synthesized, purified and characterized in house. For UC1728 the method of Hwang et al. was followed [31]. For the synthesis of UC1709 and related compounds, the methods of Tsai et al. were followed [43]. For the synthesis of UC1770, the method of Rose et al. was followed [44]. For the determination of *in vitro* potency of these inhibitors native sEH protein from rabbit liver cytosolic fraction was utilized. Protein quantification was done using the Pierce BCA assay. The potency of all inhibitors was tested using [^3^H]-*t-*DPPO as the substrate according to published methods [45,46]. For each IC_50_ determination five different concentrations of inhibitors were each tested three times. The concentration of sEH inhibitor that inhibited half of the enzyme activity is reported as the IC_50_ [45].

### Animals

Eighteen male SPF New Zealand White Rabbits (2.6-3.2 Kg) were obtained from Harlan (Indianapolis, IN) and randomly assigned to 3 groups of 6 rabbits each. Rabbits assigned to group 1 had 20 μl sterile PBS intracamerally injected in the right eye (negative control) and all other rabbits received 100 ng LPS in 20 μl of PBS [37,47-50]. Groups 2 and 3 were treated with anti-inflammatory drug or vehicle once daily on the following schedule: 24 h prior to intra-cameral LPS or PBS injection, the day of injection and 24 h post-injection. Group 2 received UC1728 in PEG400 (3 mg/kg, SC) and Group 3 received PEG400 vehicle only (0.9 mL, subcutaneously, (SC)). To limit post-procedural discomfort, systemic buprenorphine (0.03 mg/kg SC, Reckitt-Benckiser) was administered immediately prior to returning rabbits to their cages upon recovery from anesthesia, then every 6-12 h for the first 24 h and as needed for the duration of the study. Animal research followed the ARVO Statement for the Use of Animals in Ophthalmic and Vision Research; NIH Guidelines for Care and Use of Animals, and was approved by the UW-Madison Institutional Animal Care and Use Committee.

### Induction of uveitis

Uveitis was induced by intracameral injection of 100 ng of *E. coli* LPS (Sigma #L4391, St. Louis, MO) in 20 μl PBS (Mediatech, Manassas, VA) into the right eye using a 29 gauge needle, while the rabbits were anesthetized with ketamine HCl (25 mg/kg, Ft. Dodge) and xylazine (2 mg/kg, Lloyd) IM. Topical proparacaine (0.5%, Akorn, Lake Forest, IL) was applied to the ocular surface prior to intraocular injections. The ocular surface was prepared for the injection procedure using a dilute solution of 5% Povidone iodine (Aurora Pharmaceutical, Northfield, MN) in 0.9% saline (Phoenix, Burlingame, CA). During anesthesia (preparation and recovery) the cornea was protected from drying by irrigation with Balanced Salt Solution (Akorn) or, following intraocular injection, the application of ocular lubricant (Refresh Tears, Allergan) or Bacitracin-Polymixin B Preservative Free Ophthalmic Ointment (Akorn). The left eye received all the pre- and post- injection treatments, but not the LPS or PBS injection and thus was used as a procedural control for ocular preparation.

### Clinical examination and scoring of inflammation

The rabbits were examined by an experienced board-certified veterinary ophthalmologist who was masked to their treatment group assignment, by slit lamp biomicroscopy (PSL Classic, Keeler, Broomall, PA) and indirect ophthalmoscopy prior to LPS injection (baseline) and then at 6 h, 24 h and 48 h post-injection. For examination of the posterior segment, the eye was dilated with topical tropicamide drops (1%, Akorn). A modification of the Hackett-McDonald scoring system, which has been previously used by members of our group, was used in this study (Supplementary Table 1) [51-53]. This scoring system has been widely used in rabbits, with modifications to include intraocular findings such as aqueous cell and flare [37] that have been used extensively by our group in toxicological studies, including those associated with intraocular inflammation, in which it has been sensitive in discriminating between subtle degrees of iris congestion, aqueous and vitreous cell and flare. In some rabbits, topical fluorescein staining of the eye was also conducted (Ful-Glo strips, Akorn, in balanced salt solution), when corneal epithelial defects were observed or suspected. In order to minimize invasive procedures, we did not sample the aqueous for drug and protein concentrations at each time point, as sampling itself results in increased protein and could allow additional drug to enter the eye.

### Sampling

At 48 h post LPS injection, the rabbits were anesthetized with ketamine/xylazine prior to euthanasia by intravenous administration of sodium pentobarbital (Beuthanasia, Schering Plough/Merck, Kenilworth, NJ). Blood was collected, allowed to clot, and then centrifuged at 15,000 rpm for 5 min using an Eppendorf 5424 microfuge, and serum was collected and stored at -20 °C for analysis.

### LC/MS/MS analysis for UC1728 serum concentrations

The liquid chromatography system used for analysis was an Agilent 1200 SL liquid chromatography series (Agilent, Foster City, CA). The auto sampler was kept at 4 °C. Liquid chromatography was performed on a Supelco Ascentis Express C18 HPLC 5 cm×2.1 mm, 2.7 um column (Sigma). The column was connected to a 4000 QTrap tandem mass spectrometer (Applied Biosystems/Life Technologies, Carlsbad, CA) equipped with an electrospray source (Turbo V). The instrument was operated in negative selective reaction monitoring (SRM) mode. Individual analyte standards were infused into the mass spectrometer and SRM transitions and source parameters were optimized for UC1728. The SRM transition for UC1728 was 437.2/137.1; the transition for internal standard CUDA was 339.2/214.3.

### Statistical Analysis

Statistical analysis was carried out using Sigma Plot 11 (Systat Software, Inc., San Jose, CA) using the Kruskal-Wallis ANOVA on Ranks test. Differences were deemed to be significant if the p value was < 0.05.

## Results

### Selection of the sEH inhibitor

Several thousand sEH inhibitors have been synthesized to date [3] and the inhibitory potency of sEH inhibitors vary for each species. Therefore, we first determined the IC_50_ values of three inhibitors using a rabbit liver cytosolic extract. Of the three sEH inhibitors tested, the inhibitor UC1728 was the most potent with an IC_50_ value of 2 nM (Table 1) and was used for all subsequent studies.

### Clinical evaluation

**Figure 1:**
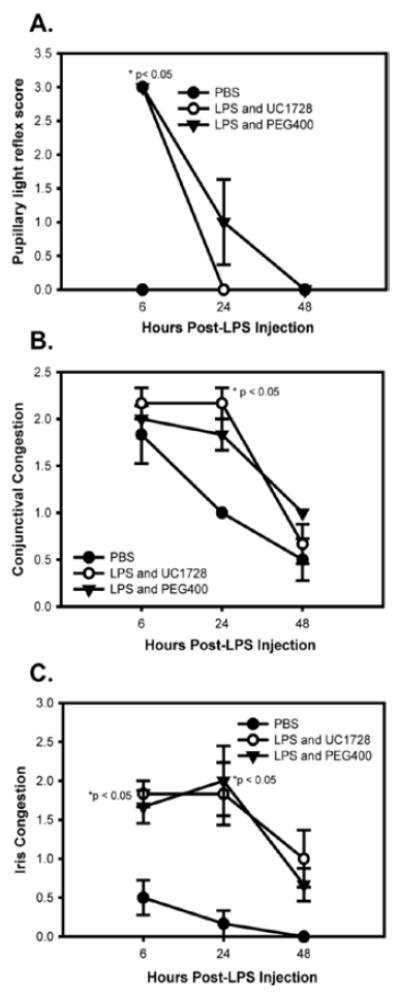
Individual uveitis parameter scores for LPS and PBS- injected eyes (mean±SEM per group). Panel A. Pupillary light reflex was assessed at baseline and 6, 24, and 48 h post-LPS injection. Scores ranged from 0, denoting a normal pupillary light reflex, to 3 which denoted a miotic pupil. In no animals was relative pupil dilation (scores 1 or 2) observed in the injected eye. Asterisks represent significant differences (p<0.05) between the LPS groups and the PBS injected group. Panel B. Conjunctival congestion scores. Asterisks represent significant differences (p<0.05) between the UC1728 and PBS groups. Scores ranged from 0 (normal) to 2+ (denoting bright red color of the bulbar and palpebral conjunctiva with peri-limbal injection involving at least 75% of the limbal circumference). Panel C. Iris congestion scores. Scores ranged from 0 (normal) to 3+ (moderate injection of secondary and tertiary iris vessels with slight iris stromal swelling). The UC1728 group was significantly different from the PBS group at 6 h post-LPS injection. At 24 h post-LPS injection, the UC1728/LPS, and PEG400/LPS groups were significantly different from the PBS group (p<0.05)

Baseline examinations conducted 24 h prior to endotoxin administration revealed normal values in all subjects. Of the several parameters evaluated in the modified Hackett-McDonald scoring system, three: pupillary light reflex (miosis), conjunctival congestion, and iris congestion were consistently increased in LPS-injected eyes as markers for LPS-induced uveitis in the rabbits. Other ocular signs associated with uveitis, including anterior chamber flare, cell and fibrin, were inconsistently observed in LPS-treated eyes. Note that fibrin is inconsistent in this model and may be independent of endotoxin concentration [37]. Figure 1 panels A, B, and C shows the scores for pupillary light reflex, conjunctival and iris congestion in LPS and PBS-treated eyes. Miotic, fixed pupils were observed in the treated eyes of both groups receiving LPS injections but the difference in pupillary light reflex scores was only significantly increased in the UC1728/LPS treated group relative to the PBS group at 6 h post-injection (1A). For conjunctival congestion, the scores for the groups receiving LPS were not significantly different from each other at any time, regardless of whether animals were treated with UC1728 or vehicle (1B). The only significant difference for conjunctival congestion was between the LPS/UC1728 group and PBS group at 24 h post-treatment. Iris congestion scores were significantly higher in eyes that received LPS compared to PBS injected eyes at 6 and 24 h post-injection (1C) but this difference was no longer statistically significant at 48 h. There were no significant differences in the scores between the LPS/UC1728, and LPS/PEG400 vehicle -treated groups at any time.

**Figure 2:**
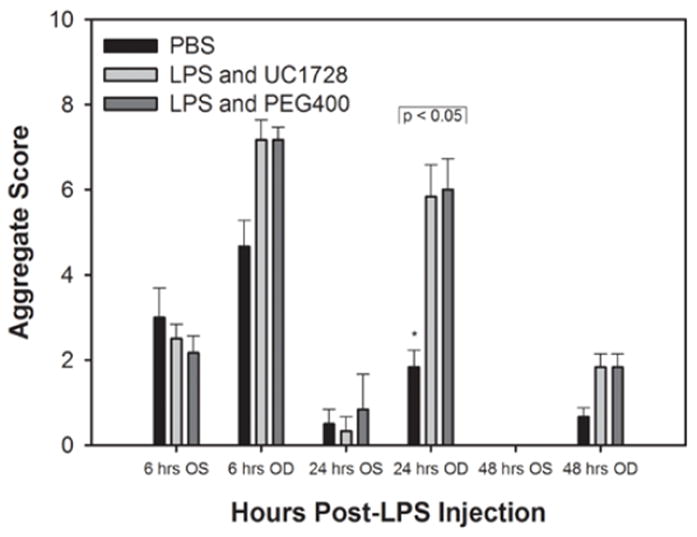
Post-injection aggregate inflammatory scores (mean±SEM per group) for non-injected, preparation only control eyes (OS) and LPS and PBS-injected eyes (OD). At 6 and 24 h post-injection, aggregate scores were significantly higher for the LPS or PBS injected eyes compared to the eyes that received preparation only. Injected eyes in the UC1728/LPS and PEG400/LPS groups had higher scores than the PBS-injected eyes, with differences that were only statistically significant at 24 h post-injection (p<0.05).

We also compared aggregate scores (obtained from addition of all scoring parameters) and these are shown in Figure 2. This figure also includes the control eyes which received the surgical preparation only (no LPS injection). At 6 h after injection, eyes that received surgical-preparation only demonstrated conjunctival congestion, with or without accompanying chemosis or ocular discharge, but these changes were mild with scores limited to 1+ for these parameters and aggregate scores that were approximately half of the aggregate scores for the LPS-treated eyes. This reaction in the preparation-only eyes diminished rapidly and was minimal by 24 h post-initiation and scores returned to baseline, normal values by 48 h. At 6 h post-LPS injection, there were no significant differences in the aggregate scores between the LPS-treated groups. At 24 h post-initiation the aggregate scores for PBS treated eyes were significantly lower than scores for the eyes injected with LPS.

Overall, these results indicate that endotoxin induced uveitis in rabbits following intracameral injection of LPS is mild and transient and that treatment with the soluble epoxide hydrolase inhibitor UC1728 had little, if any, clinical efficacy in reducing LPS-induced inflammation compared to treatment with PEG400 vehicle control.

### Serum concentrations of UC1728 at 48 hours post-treatment

To determine if therapeutic concentrations of UC1728 were likely to have been achieved we measured inhibitor concentrations in serum samples taken 48 h post-treatment at the time of euthanasia (Table 2). The LC-MS/MS method used for UC1728 has a limit of quantification of 0.2 nM [54]. The mean serum concentration of UC1728 in Group 2 rabbits was 2070±196 nM of UC1728, which is approximately 1000-fold higher than the *in vitro* IC_50_ potency value for this inhibitor (Table1). In all other groups UC1728 was undetectable as expected.

## Conclusions

Arachidonic acid metabolites have been shown to play important roles in a number of inflammatory processes and a number of arachidonic acid metabolizing enzymes are present in ocular tissues. For uveitis, there is little information available on whether EETs or metabolites such as DiHETs resulting from sEH activity, play a role but the availability of specific inhibitors makes it possible to assess the potential role of these pathways in ocular inflammation. In this work, we assessed the potential anti-inflammatory effect of an inhibitor of soluble epoxide hydrolase in a standard rabbit acute uveitis model and the results indicate that inhibition of the sEH enzyme neither enhanced nor reduced the severity of the inflammation.

The mild, transient and inconsistent nature of signs of inflammation in this particular uveitis model may have limited the ability to detect a subtle anti-inflammatory effect of UC1728. Laser flare photometry provides objective, sensitive and reproducible quantification of aqueous humor protein content and cell in clinical patients and animal models and was considered as an alternative strategy [55]. However, this method is not without limitations and there is considerable variability in values obtained from normal individuals. Degree of pupil dilation has been shown to impact values obtained by photometry and this could have confounded quantification of flare in subjects with variable degrees of mitosis in our study. A previous report comparing clinical grading of aqueous flare with aqueous flare photometry in an a rabbit endotoxin-induced uveitis model concluded that clinical slit-lamp biomicroscopy scores correlated with aqueous flare photometry values at lower grades, consistent with those observed in the current study, and indicated comparable sensitivity between these two methods for detecting subtle signs of uveitis in this model [37].

Another possible explanation for the lack of effect of UC1728 is that drug concentrations did not reach inhibitory levels. We quantified inhibitor levels in the serum of the rabbits and found the mean concentration was approximately 1,000-fold higher than the IC_50_ value, thus is it unlikely that this explains the lack of effect. Another possibility is that the inhibitor did not reach high enough concentrations in the eye, although the free inhibitor levels in blood, multiple administrations and observable breakdown in the blood-aqueous barrier observed in LPS-treated eyes make this less likely. A third possibility is that the anti-inflammatory effects of EETs and sEH are site and tissue selective. A major mechanism of action for EETs in reducing inflammation is postulated to be disruption of NFκ-B signaling [7]. These effects were initially observed in endothelial cells and later in cardiomyocytes [7,56]. The uveitis model induced by LPS similarly activates a Toll Like Receptor (TLR4) initiated signaling cascade. However, the specific mechanism initiated by LPS through TLR4 in the eye may not be identical to other tissues. Therefore target(s) of EETs or mechanisms mediated by EETs may not exist or may not interact with the same downstream signaling pathways as in endothelial cells. Consistent with this idea, Fife et al. did not observe anti-inflammatory effects in the liver tissue of mice systemically treated with LPS and sEH inhibitor [57]. Furthermore in that study the whole body knockout sEH^-/-^ mice also did not display an anti-inflammatory profile in liver tissues. However, with both chemical and genetic ablation, the plasma levels of anti- and pro-inflammatory eicosanoids were modulated in a manner that would be expected from inhibition of sEH.

It has previously been shown that DiHETs display pro-inflammatory effects by increasing the secretion of monocyte chemoattractant protein-1 (MCP-1) and inducing monocyte chemotaxis both *in vitro* and *in vivo* [58]. Therefore, one mechanism for the anti-inflammatory effects of inhibitors of sEH may be through reducing the levels of DiHETs and thus decreasing cellular infiltration at the site of inflammation. These observations may partially explain the lack of effect we saw with UC1728, since cellular infilltration was not a major feature seen in this acute LPS-induced model of inflammation.

A final possible explanation for lack of efficacy in this model is that sEH inhibitors only preserve epoxy fatty acids and do not increase their production. Thus, the concentration and rate of production of epoxy fatty acids, including EETs, may not have been sufficiently high in this model, such that their stabilization with sEH inhibitors would not have resulted in levels sufficient to prevent uveitis. This hypothesis could be tested by applying EETs and EDPs to the eye.

Although we found that inhibition of sEH did not affect LPS induced uveitis, we cannot conclude that sEH plays no role in uveitis pathophysiology. The LPS model has a very specific and restricted mechanism; activation of TLR4, which results in the downstream activation of NFκB [59], thus this model may not be appropriate for the evaluation of sEH inhibitors. It is possible that sEH may be involved in some forms of uveitis that currently available animal models do not mimic. Overexpression of sEH using gene delivery strategies or the analysis of clinical samples for the presence of sEH metabolic products might be informative as to the potential involvement of sEH and its substrates and metabolites in uveitis. Although our results showed a lack of effect, this work provides important information about the potential role, or lack thereof, of EETs and sEH metabolites in a widely used model of ocular inflammation.

## Figures and Tables

**Table 1:** Structures and potencies of inhibitors tested on rabbit liver sEH.

Name, synonym	Structure	Rabbit IC_50_ nM	Mass (Da)
*t*-TUCB UC1728	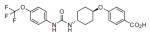	2	438.3
TUPS UC1709	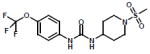	88	381.4
TPPU UC1770	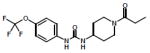	275	359.3

t-TUCB or UC1728 (*trans*-4-{4-[3-(4-Trifluoromethoxy-phenyl)-ureido]-cyclohexyloxy}-benzoic acid, TUPS or UC1709 (trifluoromethoxyphenyl-3-(1-propionylpiperidin-4-yl) urea), TPPU or UC1770 (1-methylsulfonyl-piperidin-4-yl)-3-(4-trifluoromethoxy-phenyl)-urea).

**Table 2:** SerumUC1728 Concentrations in Study Animals at 48 h Post-Treatment

Treatment Group	UC1728 Concentration(nM)* Mean±SEM
PBS	ND†
LPS + UC1728	2070.29±196.05
LPS + PEG400	ND

† ND-not detected; Mean±SEM (Standard Error of the Mean)

## Abbreviations

AA: Arachidonic Acid
DiHETS: DiHydroxyeicosatrienoic Acids
DiHOMEs: Dihydroxy Octadecamoenoate Esters
EETs: Epoxyeicosatrienoic Acids
EpOME: Monoepoxide derivatives of linoleic acid)
HETEs: Mono-hydroxy-eicosatetraenoic Acids
SC: Subcutaneously
sEH: Soluble Epoxide hydrolase
TLR: Toll Like Receptor
UC1728: trans-4-{4-[3-(4-trifluormethoxy-phenyyl)-ureido]-cyclohexyloxy}-benzoic acid
VEGF: Vascular Endothelial Growth Factor
